# Efficacy of endoscopic gastrojejunal bypass in obese Yucatan pigs: a comparative animal study

**DOI:** 10.1186/s12876-023-03000-1

**Published:** 2023-11-01

**Authors:** S Ouazzani, L Monino, L Beyer-Berjot, E Garnier, S Berdah, M Barthet, JM Gonzalez

**Affiliations:** 1 AP-HM, Department of gastroenterology, Aix-Marseille Univ, Hôpital Nord, Marseille, France; 2 Centre d’Enseignement et de Recherche Chirurgical, Aix-Marseille Univ, Marseille, France; 3 Department of gastroenterology and Hepatopancreatology, ULB, HUB, Erasme Hospital, Brussels, Belgium; 4 AP-HM, Department of digestive surgery, Aix-Marseille Univ, Hôpital Nord, Marseille, France

**Keywords:** Obesity, Bariatric surgery, Gastric bypass, Metabolic endoscopy, NOTES

## Abstract

**Background:**

Natural orifice transluminal endoscopy surgery (NOTES) gastrojejunal anastomosis (GJA) with duodenal exclusion (DE) could be used as a less invasive alternative to surgical gastric bypass. The aim of this study was to compare the efficacy and safety of both methods for bariatric purpose.

**Methods:**

This was a prospective, experimental and comparative study on 27 obese living pigs, comparing 4 groups: GJA alone (group 1, G1), GJA + DE (group 2, G2), surgical gastric bypass (group 3, G3), control group (group 4, G4). GJA was endoscopically performed, using NOTES technic and LAMS, while DE was performed surgically for limb length selection. Animals were followed for 3 months. Primary outcome included technical success and weight change, while secondary endpoints included the rate of perioperative mortality and morbidity, histological anastomosis analysis and biological analysis.

**Results:**

Technical success was 100% in each intervention group. No death related to endoscopic procedures occurred in the endoscopic groups, while early mortality (< 1 month) was 57,1% in the surgical group, all due to anastomotic dehiscence. At 3 months, compared to baseline, mean weight change was + 3,1% in G1 (p = 0,46); -14,9% in G2 (p = 0,17); +5,6% in G3 (p = 0,38) and + 25% in G4 (p = 0,029). Histopathological analysis of endoscopic GJA showed complete fusion of different layers without leak or abscess.

**Conclusions:**

Endoscopic GJA with DE provides the efficacy of bypass on weight control in an animal model. Next steps consist of the development of devices to perform exclusively endoscopically limb length selection and DE.

**Supplementary Information:**

The online version contains supplementary material available at 10.1186/s12876-023-03000-1.

## Background

For 50 years, obesity has been dramatically rising, exceeding 35% of the United States population, and also increasing in other developed and less developed countries [1, 2]. Bariatric surgery offers a substantial and sustained weight loss, and comorbidities improvement [1–3]. Roux-en-Y gastric bypass (RYGBP) is one of the most effective procedure with a perioperative mortality rate ranging from 0,3 to 4%, with an overall complication rate reaching 17% [4, 5].

For these reasons, development of less invasive and reversible techniques are emerging as natural orifice transluminal endoscopic surgery (NOTES), which could reduce morbidity and mortality [6]. These new approaches could also represent a bridge for very high-risk patients who would be excluded for classic bariatric interventions [7]. Minipig breeds, as Yucatan, Ossabaw and Göttingen, have reduced size at the adulthood, and are easier to handle, if obesity is induced [8–10]. As exclusive endoscopic ultrasound (EUS)-guided gastroenteric anastomosis based on lumen-apposing metal stent (LAMS) could not be adapted to bariatric endoscopic bypass because of the lack of measurement of the bypassed limb, our team developed a NOTES procedure in a swine model [11, 12] This NOTES procedure for endoscopic bypass was also performed and published in human beings [13].

The aim of the present experimental animal study was to compare the safety, the efficacy and the metabolic consequences of an exclusively endoscopic bypass (consisting in a GJA associated or not to a duodenal exclusion), to classic surgical RYGP and control groups, in obese Yucatan pigs.

## Methods

### Study design

This was a prospective experimental comparative animal study conducted at the Center for Surgical Education and Research (CERC – Centre d’Enseignement et Recherche en Chirurgie) of the Faculty of Medicine North at the Aix-Marseille University (France). The study design, the care and the handling of animals were approved by the institutional review board of the Aix-Marseille University (Ethical comitee #14) and French Authorities (Ministère de l’Enseignement et de la Recherche, authorization APAFIS #22,017,033,011,503,900 v3).

Pigs were supplied by INRA (Institut National de la Recherche Agronomique, Rennes, France). All applicable institutional and/or national guidelines for the care and use of animals were followed. Four groups were predefined: group 1 (G1), consisting of endoscopic bypass with GJA without pyloric closure; group 2 (G2) consisting of endoscopic bypass with GJA and surgical duodenal exclusion; group 3 (G3) consisting of surgical RYGB; and group 4 (G4) consisting of a control group, without any intervention.

The endoscopic procedures were performed by two experts in therapeutic endoscopy (MB and J-MG), while surgical bypass procedures were performed by two surgeons, experienced in bariatric surgery (SB and LB).

### Animals handling and anesthesia protocol

All animals were obese Yucatan pigs, aged 12 months, rendered obese with insulin-resistance after hypercaloric alimentation since they were 9 months old at the INRA.

All animals arrived 5 days before the procedures at the CERC for acclimation and were housed individually. They received water and pig chow diet. All of them received a 14 cm double lumen (20 Gauge) venous central catheter (VCC) (Arrow, Kendall Health Care Products, Mansfield, EU) 2 days before intervention, placed in the jugular vein and immediately tunnelized. The catheters were used for test meals, initially left in place in the first animals, and then removed immediately after the tests because of infection and lethal vascular complications. Feeding was stopped 24 h before endoscopic or surgical intervention.

For anesthesia induction, animals received intramuscular injections of both azaperone 1 mg/kg and ketamine 5 mg/kg. Anesthesia was maintained with continuous intravenously injection of 100 mg/h of propofol 2% and 100 micrograms/hour of remifentanil for analgesia. They were intubated and mechanically ventilated. Perioperative antibiotic prophylaxis was administrated by intravenously injection of Cefoxitine 2 g and continued twice daily during postoperative period. Each animal had close monitoring with heart rate and oxygen saturation during the procedure, performed in supine position in the three intervention groups.

### Endoscopic procedures

**Fig. 1:**
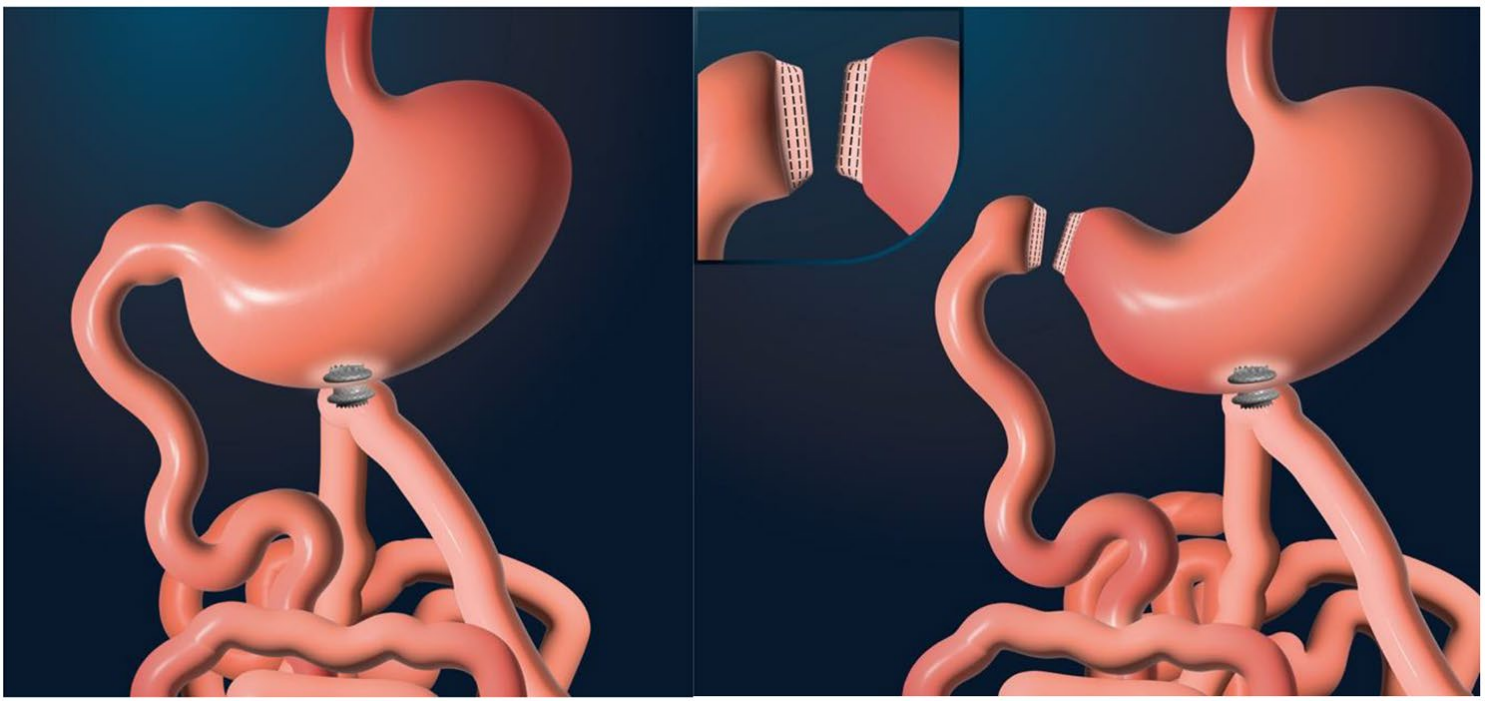
Schematic description of endoscopic procedures (groups 1 and 2). (**a**). GJA without duodenal exclusion (Group 1). (**b**). GJA with surgical duodenal exclusion (Group 2)

Procedures of GJA creation were previously described by our team [11, 12]. Briefly, a dual-channel video gastroscope (3.7 and 2.8 mm; XGIF-2T180H; Olympus Europe, Hamburg, Germany) was used and the following procedures were performed in the 2 first endoscopic groups. For this study, measurement of the bypassed limb length was done surgically despite the GJA was done endoscopically:

1. *Mini laparotomy for limb selection.* A surgical median laparotomy was performed by the surgeons and limb selection at 300 cm from the pylorus.
2. *Endoscopic gastric parietal incision*, performed in the horizontal portion of the anterior preantral zone, away from the small and large curvature, using a Hook Knife (Olympus, Japan).
3. Access to the peritoneal cavity, followed by *prehension of the jejunal loop*, presented by the surgeon, using a twin grasper forceps (OTSC Twin Grasper; OVESCO), and a 0.035” guidewire was inserted in the limb, after a parietal puncture with a 19 Gauge needle.
4. *LAMS* (Boston Scientific, USA) *insertion* over the wire, *and deployment.* First the distal flange was into the jejunum, then the limb was then gently pulled into the gastric lumen, using both distal flange of the stent and the twin-grasper, followed by proximal flange into the stomach (Fig. 1a).

In the second group, to mimic a surgical bypass-like malabsorptive effect, a laparotomic duodenal exclusion was surgically performed with a stapler placed at the level of the genu superius (Fig. 1b).

### Surgical procedure

Surgical bypass was performed following standard way, using conventional laparotomy equipment. A classic RYGB was created, with a 300 cm bypass limb, a pancreatobiliary limb and a gastric pouch. The 300 cm length was chosen because the small bowel length in a pig is twice as long as in humans; therefore, we decided to double the length of the alimentary loop to mimic the same ratio than applied in human’ by-passes.

### Follow-up and euthanasia

After the procedure, each animal was clinically observed during a period of 3 months. They were maintained nihil per os during the first 24 h, followed by water at the 1st postoperative day (POD) and finally progressive re-feeding at POD3 (they received a quarter of the usual pig chow for 48 h, then half pig chow for 48 h, followed by 3 quarters for 48 h, before being fed normally until the end of follow-up). All animals received a standardized meal. Antibiotic prophylaxis was continued for three days. Different clinical parameters (overall behavior, food intake, temperature, pain, bowel and urinary functions) were monitored intensively the first 2POD, and twice daily after. Intramuscular injection of tramadol 100 mg was administered twice daily during the three first days, and in case of signs of pain. Failure to eat, vocalization and teeth grinding were considered as signs of pain.

**Fig. 2:**
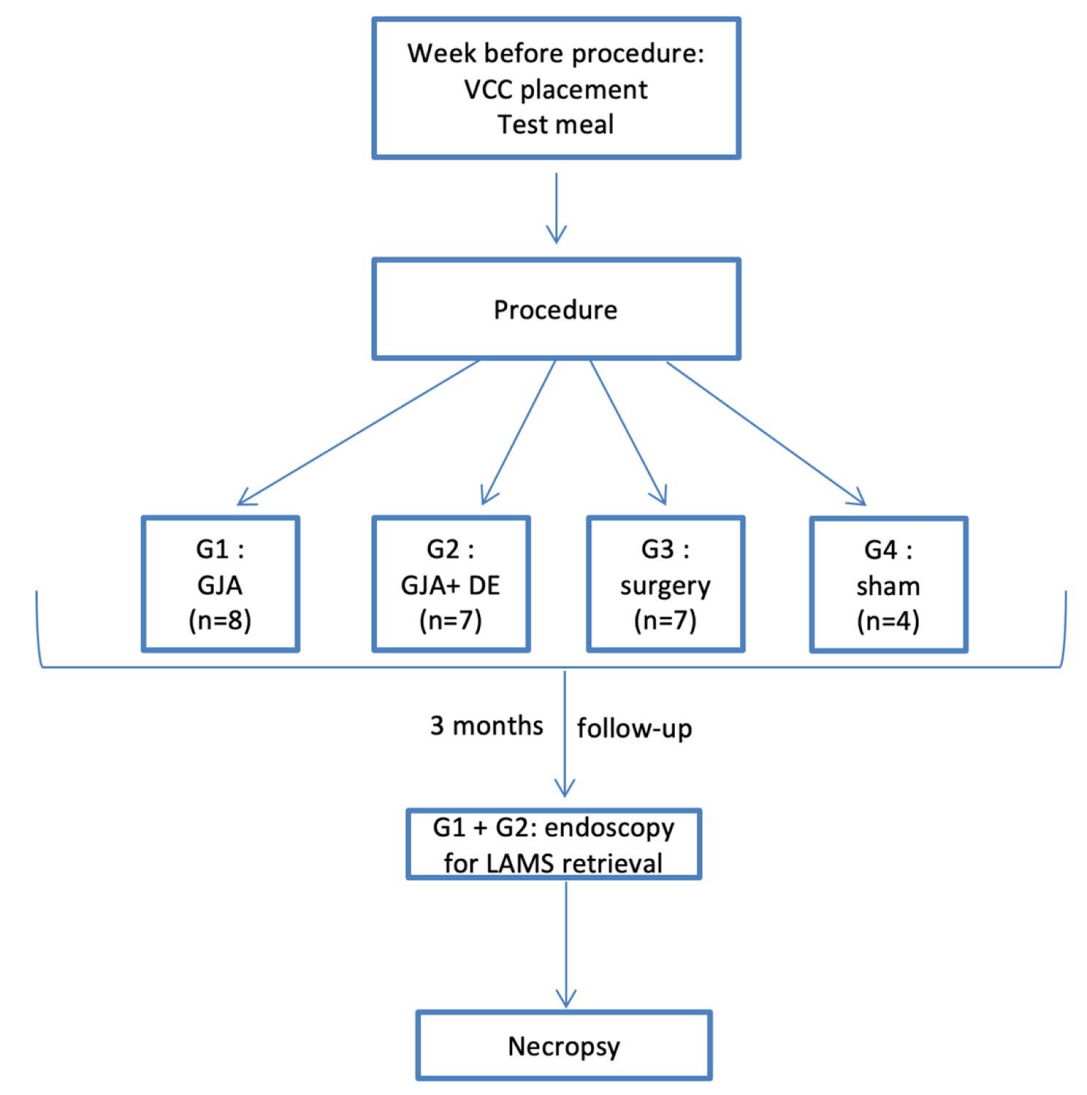
Study flowchart

At the end of follow-up, animals from endoscopic G1 and G2 had endoscopic LAMS retrieval, followed by GJA evaluation through laparotomy. Then all survival animals were sacrificed with administration of a lethal dose of pentobarbital. Necropsies were performed among animals with premature death, and all surviving animals from G1 and G2, allowing macroscopical and microscopical anastomosis evaluation. The study flowchart is summarized in Fig. 2.

### Metabolic and hormonal assessment

For biological assessment, all samples were taken in CERC, through the VCC, and plasmatic dosages were performed in the research Unit UMR S 1260 of the faculty of Medicine, University Aix-Marseille.

A test meal was performed in each animal of all groups, for following molecules: glucose, insulin, peptide YY (PYY), xylose, FGF-19, FGF-21, GLP-1 and ghrelin.

Before the procedure and after 12 h of fasting, all animals had a meal containing 1925 kcal for 30 min. Blood samples were collected 15 min before the meal (t0), and then dynamic dosages were performed at 30, 60 and 120 min after the meal, except for the ghrelin (only a fasting sample was performed).

After the end of the FU, survival animals had a second test meal, under exactly same conditions as before procedure.

### Endpoints and outcomes

The primary endpoints of this study were the weight after a follow-up of three months in each animal group. Secondary endpoints included the rate of perioperative mortality and morbidity, histological anastomosis analysis and biological analysis.

### Statistical analysis

Descriptive statistical analysis of quantitative variables were expressed as median (with range) or mean (with standard deviation), while qualitative variables are expressed as a percentage. Non-parametric Mann-Witney tests were used to determine the significance of difference between 2 groups means and non-parametric Kruskal-Wallis test was performed for weight comparison between multiple groups. All tests were two-sided, with significance level determined at 5%. All statistical analyses were performed using SPSS.

## Results

### Animal characteristics

A total of 27 pigs were included in four groups as following: 8 in the endoscopic GJA without pyloric closure (G1), 7 in the complete endoscopic bypass (G2), 7 in the surgical bypass (G3) and 5 in the control group (G4). At the baseline, the mean weights in each group were respectively 62,7 kg (± 4,2); 61,8 kg (± 2,46); 65 kg (± 4,1) and 64 kg (± 4), without statistical difference (p-value = 0,42, with p > 0,05 for each comparison).

### Technical success and adverse events

GJA with endoscopic stent placement was performed in a mean time of 24 min (± 10 min) in G1 and G2, while the surgery was performed in a mean time of 116 min (± 36 min) in G3 (p < 0,001).

In endoscopic groups, there were 2 intra-operative adverse events: one proximal flange slipping into the peritoneum and one forceps dysfunction which occurred during limb traction into the stomach. In both cases, the events were endoscopically successfully managed, without any clinical consequences: in the first, the proximal flange was replaced into the stomach using a rat-tooth forceps while in the latter, a second LAMS was used to complete the GJA.

In each interventional group, the final technical success was 100%.

2/8 pigs died (25%) in G1, both of them after 79 days of follow-up, 2/7 in G2 (28,5%) after a median time of 47,5 days (R:10–85), 4/7 (57,1%) G3 after a median time of 3 days (R:2–14) and 1/5 (20%) in G4, after one month of F-U.

The causes of death were respectively: VCC infection (2/2) in G1, anesthesia-related death (1/2) and undetermined (1/2) in G2, anastomosis leakage (4/4) in G3 and undetermined in the last group (during transfer to other facility). In endoscopic groups, a necropsy was performed in all four animals with premature death, and all had an intact GJA, without any leakage or ongoing or previous peritonitis.

### Weight evolution

At the end of follow-up, the mean weights in each group were as following: 64,7 ± 4,1 kg in G1 (mean weight gain of 3,1%; p = 0,46); 53,8 ± 10,5 kg in G2 (mean weight reduction of 14,9%; p = 0,17); 68,7 ± 0,49 kg in G3 (mean weight gain of 5,6%; p = 0,38); 85,2 ± 1,14 kg (mean weight gain of 25%; p = 0,029).

The comparison of mean weights between endoscopic groups showed greater efficacy of the combination of GJA and DE over GJA alone (p = 0,026). Global results are presented in Table 1, comparison between the endoscopic groups is presented in Table 2.

**Table 1:** Comparison of all groups

	Group 1 (GJA alone) N = 6/8	Groupe 2 (GJA + DE) N =6/7	Group 3 (Surgery) N = 3/7	Group 4 (Control) N = 4/5
Baseline				
- Mean weight (± SD)	62,7 ± 4,2	61,8 ± 2,4	65 ± 4,1	64 ± 4
Technical efficacy	100% (6/6 or 8/8)	100% (6/6 or 7/7)	100% (3/3 or 7/7)	NA
Adverse events				
- Mortality - Causes of death	2/8 (25%) VVC infection (2)	2/7 (28,5%) Anesthesia related (1) Undetermined (1)	4/7 (57,1%) AL (4)	1/5 (20%) Undetermined (1)
End of F-U:				
- Mean weight (± SD) - % Total eight variation (mean)	64,7 ± 4,1 + 3,1%	53,8 ± 10,5 -14,9%	68,7 ± 0,5 + 5,6%	85,2 ± 1,15 + 25%

AL: anastomosis leakage; DE: duodenal exclusion; F-U: follow-up; GJA: gastrojejunal anastomosis; SD: standard deviation; VCC: venous central catheter

**Table 2:** Comparison of endoscopic groups

	Group 1 (GJA alone) N = 6/8	Groupe 2 (GJA + DE) N =6/7	P-value
Adverse events			
- GJA migration	0% (0/6)	50% (3/6)	NA
End of F-U:			
- Mean weight (± SD) - % Total eight variation (mean)	64,7 ± 4,1 + 3,1%	53,8 ± 10,5 -14,9%	P = 0,025

DE: duodenal exclusion; F-U: follow-up; GJA: gastrojejunal anastomosis; SD: standard deviation

**Fig. 3:**
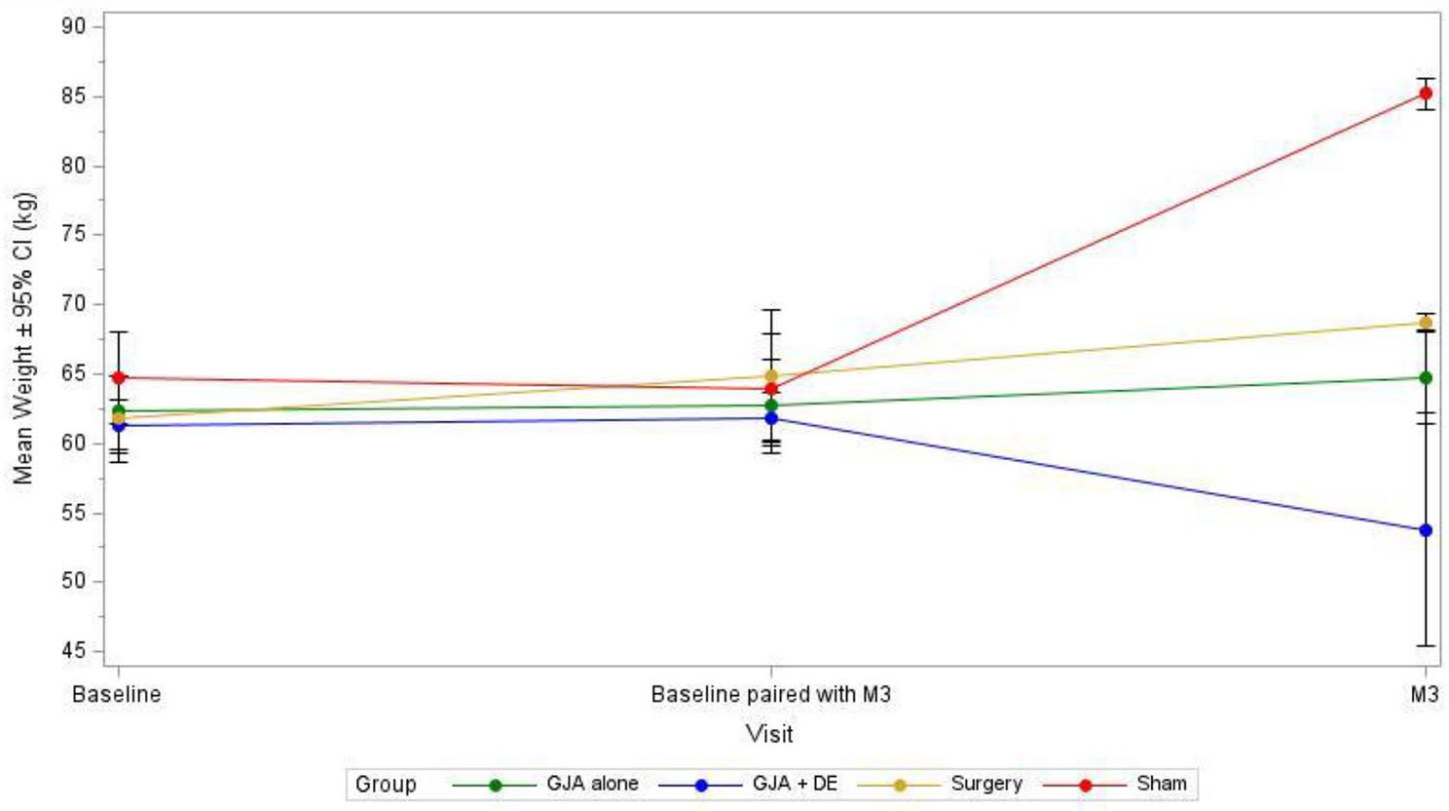
Evolution of weights in each group

The comparison of weights evolution is showed in Fig. 3.

### Post-LAMS removal follow-up, necropsy and anastomosis histological evaluation

During endoscopic evaluation (death or at 3 months), all animals in the G1 had the LAMS in place, while it migrated in 3/6 (50%) in group 2, but with still patent GJA despite a narrowing anastomosis without occlusive symptoms. All LAMS were removed without technical difficulties.

**Fig. 4:**
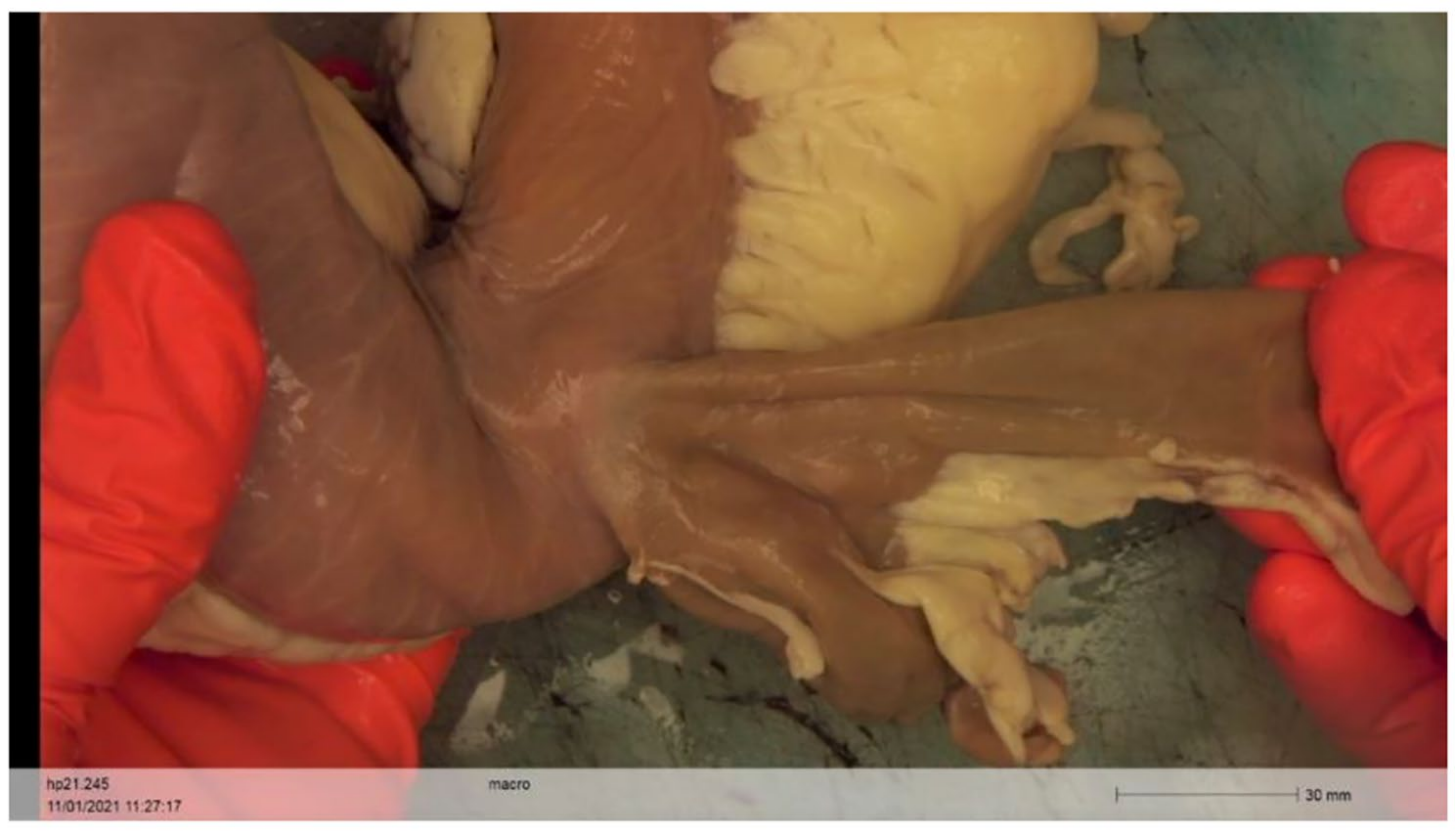
Macroscopic aspect of GJA: Picture of the gastrojejunal anastomosis (ex vivo bloc) at 3 months (after the LAMS retrieval), with stomach at the left-side and the jejunum at the right-side

**Fig. 5:**
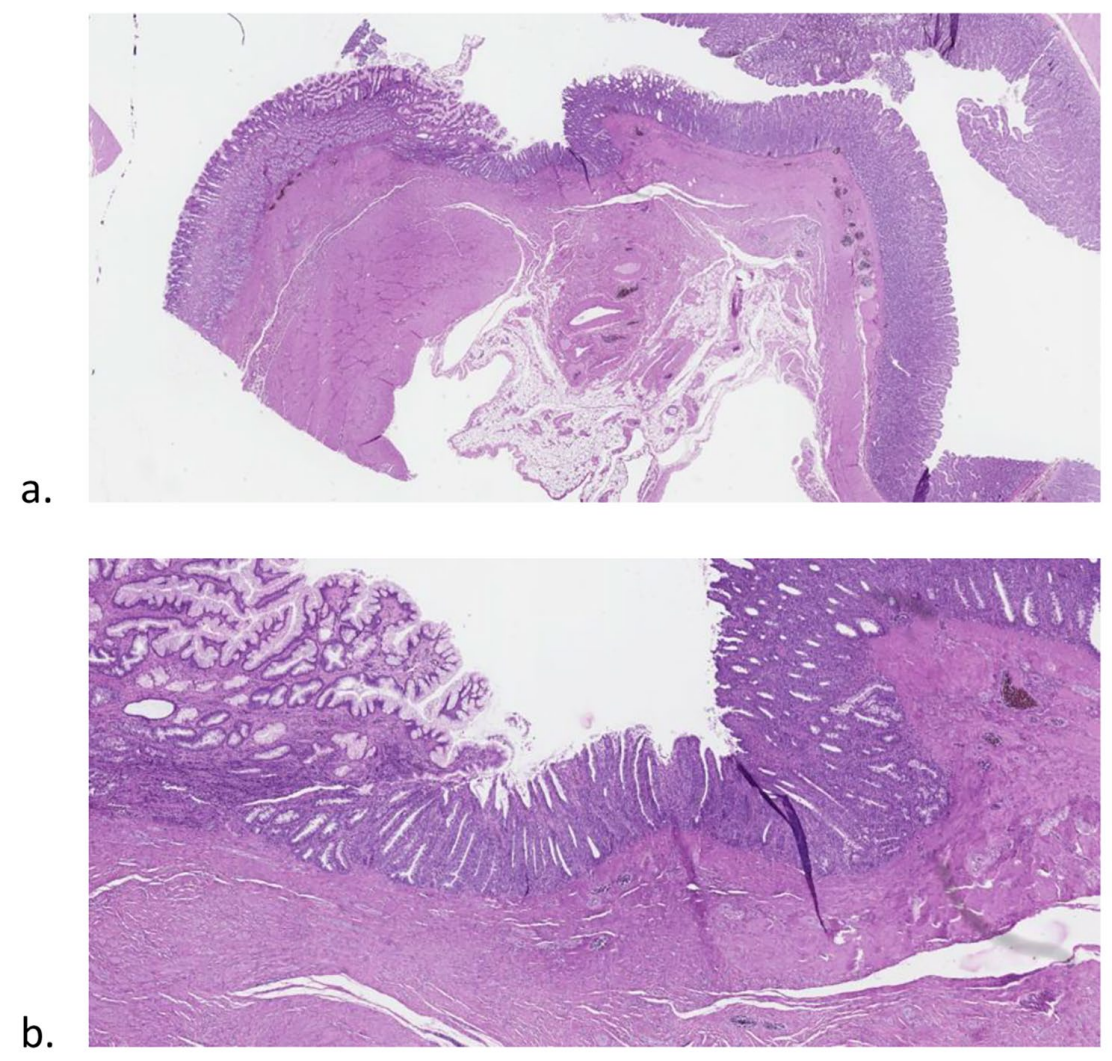
Histological assessment of the GJA: (**a**) Fusion of the gastric (left side of the picture) and the jejunal (right side of the picture) mucosal layers. (**b**) Same picture magnification and showing mild inflammatory changes in the central anastomotic areas

During necropsies, all endoscopic GJAs appeared healed macroscopically (Fig. 4), without any sign of perforation, fissure, abscess or peritonitis signs (Fig. 5). At histological level, we observed a complete fusion of mucosal, submucosal and muscular layers at the location of GJA (Fig. 5).

### Metabolic and hormonal evaluation

Because of technical issues related to samples hemolysis and coagulation, majority of biological evaluation could not be performed. Only interesting biological results are showed in this part:

- Glucose: In G2, mean fasting glycemia was reduced after intervention (1,12 vs 0,74; p = 0,095), as at 30’ (1,18 vs. 0,81; 0,29), 60’ (1,05 vs. 0,89; p = 0,49) and 120’ (1,05 vs. 0,89; p = 0,18).
- Insulin: In endoscopic groups (G1 and G2), fasting insulinemia decreased after procedure, (G1: 154,9 vs. 62,2 p = 0,34; G2: 218,4 vs. 151,1; p = 0,39).

All interpretable results are showed in Table 3. All other results are illustrated in tables included in supplementary data.

**Table 3:** Metabolic and hormonal evaluation

	G1 B vs. at 3 M (p-value)	G2 B vs. at 3 M (p-value)	G3 B vs. at 3 M (p-value)	G4 B vs. at 3 M (p-value)
Glucose				
- Fasting - 30’ - 60’ - 120’	NA 0,85 vs. 1,23 (0,29) 0,87 vs. 1,23 (0,49) 0,98 vs. 1,12 (1)	1,12 vs. 0,74 (0,25) 1,18 vs. 0,81 (0,11) 1,05 vs. 0,89 (0,47) 1,05 vs. 0,86 (0,19)	NA NA NA NA	0,72 vs. 0,67 (0,5) 0,72 vs. 0,68 (0,7) 0,71 vs. 0,77 (0,82) 0,78 vs. 0,79 (1)
Insulin				
- Fasting - 30’ - 60’ - 120’	154,9 vs. 62,2 (0,34) 243,6 vs. 174 (0,9) 209,1 vs. 124 (0,41) 111,9 vs. 122,5 (0,86)	218,4 vs. 151,1 (0,39) 212,4 vs. 148,2 (1) 181,6 vs. 222,9 (0,73) 54,4 vs. 240,6 (0,23)	NA NA NA NA	86,8 vs. 83,8 (0,7) 105,7 vs. 92,9 (1) 147,9 vs. 122,6 (0,7) 149,6 vs. 118,9 (0,7)

B: at baseline; 3 M: after 3 months of F-U; NA: non-applicable

## Conclusions

Despite the recognized place for bariatric surgery, emerging endoscopic bariatric techniques had been developed in last decade, initially used as bridge to surgery, with possible reversibility. Drawbacks consist of limited long-term weight loss maintain for balloon device [14–16], limited patient acceptability for aspiration devices [15] and mixed results for endoscopic gastroplasty in meta-analysis and when the latter are compared to FDA thresholds [16–19]. The efficacy of RYGB surgery is partially based on food diversion from proximal intestine (including malabsorptive effect and global energy homeostasis alteration) [20]. Based on these mechanisms and with the recent development of exclusive endoscopic gastro-jejunal anastomosis [11], we decided to develop a new endoscopic malabsorptive technique.

In this study, bypassing the proximal small intestine by creating of a GJA, avoided weight gain in animals compared to the control group (G4) (25% of weight gain). Indeed, weight was stabilized in G1 (GJA alone), which was equivalent to G3 (surgical RYGB), and decreased in G2 (GJA + DE) (15% of weight loss). The comparison of the two endoscopic groups also showed that DE in addition to GJA had more effect on weight change than GJA alone. Surprisingly, RYGB was less effective than endoscopic bypass, probably because of the bias due to the small number of animals who survived and were analyzed. Unfortunately, a majority of blood specimens were uninterpretable (coagulation) for several reasons, related to the race of animals (procoagulant state in Yucatan) [21], catheter issues and lab organization. Thus, the clinical outcomes could not be confronted with biological effect on gut-peptides activity and glucoses homeostasis. Globally, we found an improvement in glycemic profile in the group 2 (GJA + DE), an improvement in fasting insulinemia in both endoscopic groups, but without reaching the statistical significance. Nevertheless, these findings highlight the need for gastric outlet closure and the diversion of food from proximal small intestine to optimize the metabolic effect. One of the main sources of morbidity and mortality in bariatric surgery is the occurrence of leakages, despite when the procedures are performed by expert hands. This was translated in our surgical group (4 deaths by leakage), as already reported in previous studies in porcine models [22–24]. These outcomes contrast with those of endoscopic groups, in which no death was related to endoscopic procedure, especially leakage, as confirmed by all the necropsies performed for premature death. These 4 deaths in G1 and G2 were related to VCC complication, a non-rare complication as previously reported [24], to anesthetic complication during the endoscopy performed at the end of the F-U (at day 85). One of the limitations we have to recognize is that the study is not controlled. In fact, due to our facility capability, the size and weight of the animals (kept 3 months which is long for an animal study) and the availability of the devices developed and provided by Boston Scientific’s engineers, we had to complete the groups separately. However, all the animals were conditioned and prepared in the same laboratory (Inserm institute, Rennes) that has demonstrated in several studies the reproducibility of the model. Moreover, the were no differences in the baseline pigs’ characteristics.

LAMS migrations with partial narrowing of the GJA was quite frequent. Interestingly, all occurred in G2 (GJA + DE), probably due to an increased hyper-pressure on the GJA induced by the DE. However, all stomachs were empty at the procedure, and despite a trend towards anastomotic stricture, there were no clinical consequences such as occlusive syndrome. Consequently, we adapted our protocol by performing GJA and DE during two different steps separated by two weeks. In another hand, all remaining LAMS were removed without technical difficulty and the histology analysis showed healed anastomosis.

In conclusion, our experience demonstrates the safety of endoscopic GJA with DE compared to RYGB in Yucatan obese and fragile pigs. It also suggests the efficacy on weight change compared to a control group. The next steps are the development of new endoscopic devices for performing this procedure endoscopically only, including DE and limb selection.

### Electronic supplementary material

Below is the link to the electronic supplementary material.

Supplementary Material 1

## Data Availability

The datasets during and/or analysed during the current study available from the corresponding author on reasonable request.

## Abbreviations

CERC: Centre d’Enseignement et Recherche en Chirurgie
DE: Duodenal exclusion
EUS: Endoscopic ultrasonography
F-U: Follow-up
GJA: Gastrojejunal anastomosis
INRA: Institut National de la Recherche Agronomique
LAMS: Lumen-apposing metal stent
NOTES: Natural orifice transluminal endoscopic surgery
POD: Postoperative day
PYY: Peptide YY
RYGBP: Roux-en-Y gastric bypass
VCC: Venous central catheter
