# Supplementary material for: Efficacy of endoscopic gastrojejunal bypass in obese Yucatan pigs: a comparative animal study
Source: BMC Gastroenterol. 2023 Nov 1;23:375. doi: 10.1186/s12876-023-03000-1 (PMC10621135; doi:10.1186/s12876-023-03000-1)
Supplement: Supplementary file 1 — Supplementary Material 1 [file 12876_2023_3000_MOESM1_ESM.docx]

Table 1S: Glucose evaluation.

Group 1:

|  | **P1**  **B vs at 3M** | **P2**  **B vs at 3M** | **P3**  **B vs at 3M** | **P4**  **B vs at 3M** | **P5**  **B vs at 3M** | **P6**  **B vs at 3M** | **P7**  **B vs at 3M** |
| --- | --- | --- | --- | --- | --- | --- | --- |
| **Pre vs post**   - **Fasting** - **30’** - **60’** - **120’** | 0,67 vs NA  0,55 vs NA  0,89 vs NA  1,01 vs NA | 1,15 vs NA  1,07 vs NA  0,8 vs NA  0,98 vs NA | 0,9 vs NA  0,95 vs NA  0,96 vs NA  0,96 vs NA | NA vs NA  NA vs 1,24  NA vs 1,16  NA vs 1,14 | NA vs NA  NA vs 0,99  NA vs 0,95  NA vs 0,91 | 0,54 vs 1,23  0,83 vs 0,69  0,81 vs 0,71  NA vs 0,83 | 0,89 vs NA  0,82 vs 1,77  NA vs 1,02  NA vs 1,41 |

B: at baseline; 3M: after 3 months of F-U; NA: non-applicable

Group 2:

|  | **P1**  **B vs at 3M** | **P2**  **B vs at 3M** | **P3**  **B vs at 3M** | **P4**  **B vs at 3M** | **P5**  **B vs at 3M** |
| --- | --- | --- | --- | --- | --- |
| **Pre vs post**   - **Fasting** - **30’** - **60’** - **120’** | 1,24 vs 0,66  NA vs 0,67  0,86 vs 0,67  2,55 vs 0,82 | 1,76 vs 0,71  1,36 vs 0,69  1,54 vs 0,99  1,88 vs 0,77 | 1,05 vs 0,84  1,21 vs 0,88  0,95 vs 0,68  1,23 vs 0,85 | 0,95 vs NA  0,97 vs NA  0,97 vs NA  0,83 vs NA | 0,63 vs NA  NA vs 1,00  NA vs 1,24  NA vs 1,01 |

B: at baseline; 3M: after 3 months of F-U; NA: non-applicable

Group 3:

|  | **P1**  **B vs at 3M** | **P2**  **B vs at 3M** | **P3**  **B vs at 3M** |
| --- | --- | --- | --- |
| **Pre vs post**   - **Fasting** - **30’** - **60’** - **120’** | 0,88 vs NA  0,78 vs NA  0,82 vs NA  1,10 vs NA | NA vs 0,79  NA vs 1,02  NA vs 0,96  NA vs 1,05 | NA vs 0,74  NA vs 0,96  NA vs 0,84  NA vs 1,24 |

B: at baseline; 3M: after 3 months of F-U; NA: non-applicable

Group 4:

|  | **P1**  **B vs at 3M** | **P2**  **B vs at 3M** | **P3**  **B vs at 3M** | **P4**  **B vs at 3M** |
| --- | --- | --- | --- | --- |
| **Pre vs post**   - **Fasting** - **30’** - **60’** - **120’** | NA vs 0,62  NA vs 0,62  NA vs 0,78  NA vs 0,85 | 0,65 vs 0,82  0,68 vs 0,88  0,73 vs 0,92  0,82 vs 0,80 | 0,68 vs 0,58  0,69 vs 0,55  0,62 vs 0,61  0,75 vs 0,74 | 0,82 vs NA  0,79 vs NA  0,78 vs NA  0,76 vs NA |

B: at baseline; 3M: after 3 months of F-U; NA: non-applicable

Table 2S: Insulin evaluation.

Group 1:

|  | **P1**  **B vs at 3M** | **P2**  **B vs at 3M** | **P3**  **B vs at 3M** | **P4**  **B vs at 3M** | **P5**  **B vs at 3M** | **P6**  **B vs at 3M** | **P7**  **B vs at 3M** |
| --- | --- | --- | --- | --- | --- | --- | --- |
| **Pre vs post**   - **Fasting** - **30’** - **60’** - **120’** | 139,13 vs NA  84,04 vs NA  95,05 vs NA  125,10 vs NA | 96,27 vs NA  108,4 vs NA  119,51 vs NA  52,34 vs NA | 361,01 vs NA  321,86 vs NA  122,4 vs NA  158,38 vs NA | NA vs 61,35  NA vs 354,15  NA vs 199,51  NA vs 197,96 | NA vs 72,13  NA vs 119,45  NA vs 142,66  NA vs 102,86 | NA vs 79,85  324,8 vs 126,36  365,97 vs 85,9  NA vs 136,33 | 23,03 vs 35,63  379,11 vs 95,91  342,74 vs 67,93  NA vs 52,64 |

B: at baseline; 3M: after 3 months of F-U; NA: non-applicable

Group 2:

|  | **P1**  **B vs at 3M** | **P2**  **B vs at 3M** | **P3**  **B vs at 3M** | **P4**  **B vs at 3M** | **P5**  **B vs at 3M** |
| --- | --- | --- | --- | --- | --- |
| **Pre vs post**   - **Fasting** - **30’** - **60’** - **120’** | 45,1 vs 20,21  33,11 vs 103,63  28,71 vs 157,52  15,63 vs 137,75 | 65,95 vs NA  27,97 vs 6,62  47,68 vs 44,87  NA vs 18,7 | 140,44 vs 40,03  91,91 vs 39,05  80,02 vs 74,61  95,42 vs 110,98 | 80,25 vs NA  73,46 vs NA  60,39 vs NA  52,25 vs NA | 760,11 vs 392,95  835,64 vs 587,41  691,14 vs 614,53  NA vs 694,81 |

B: at baseline; 3M: after 3 months of F-U; NA: non-applicable

Group 3:

|  | **P1**  **B vs at 3M** | **P2**  **B vs at 3M** | **P3**  **B vs at 3M** |
| --- | --- | --- | --- |
| **Pre vs post**   - **Fasting** - **30’** - **60’** - **120’** | 40,25 vs NA  41,67 vs NA  36,04 vs NA  35,73 vs NA | NA vs 229,23  NA vs 193,84  NA vs 57,55  NA vs 243,36 | NA vs 151,72  NA vs 103,32  NA vs 69,62  NA vs 287,19 |

B: at baseline; 3M: after 3 months of F-U; NA: non-applicable

Group 4:

|  | **P1**  **B vs at 3M** | **P2**  **B vs at 3M** | **P3**  **B vs at 3M** | **P4**  **B vs at 3M** |
| --- | --- | --- | --- | --- |
| **Pre vs post**   - **Fasting** - **30’** - **60’** - **120’** | NA vs 83,95  NA vs 106,07  NA vs 137,72  NA vs 108,03 | 94,17 vs 88,94  158,92 vs 106,31  173,81 vs 166,32  175,87 vs 184,26 | 56,99 vs 78,51  55,66 vs 66,38  149,14 vs 63,78  132,79 vs 64,5 | 109,08 vs NA  102,55 vs NA  120,88 vs NA  140,14 vs NA |

B: at baseline; 3M: after 3 months of F-U; NA: non-applicable

Table 3S: PYY evaluation.

Group 1:

|  | **P1**  **B vs at 3M** | **P2**  **B vs at 3M** | **P3**  **B vs at 3M** | **P4**  **B vs at 3M** | **P5**  **B vs at 3M** | **P6**  **B vs at 3M** | **P7**  **B vs at 3M** |
| --- | --- | --- | --- | --- | --- | --- | --- |
| **Pre vs post**   - **Fasting** - **30’** - **60’** - **120’** | \| 186,9 vs NA \| \| --- \| \| 208,1 vs NA \| \| 261,5 vs NA \| \| 222,3 vs NA \| | \| 165,7 vs NA \| \| --- \| \| 180,7 vs NA \| \| 189,9 vs NA \| \| 154,5 vs NA \| | \| 179,1 vs NA \| \| --- \| \| 197,5 vs NA \| \| 176,4 vs NA \| \| 192,4 vs NA \| | NA vs 196,7  NA vs 189,9  NA vs 209,7  NA vs 201,2 | \| NA vs 260,8 \| \| --- \| \| NA vs 204,5 \| \| NA vs 197,7 \| \| NA vs 216,2 \| | \| 106,2 vs 154,6 \| \| --- \| \| 168,4 vs 121,2 \| \| 158,4 vs NA \| \| NA vs 148,2 \| | \| 154,4 vs 118,3 \| \| --- \| \| 135,9 vs 119,2 \| \| 153,6 vs 128,7 \| \| ND vs 111,4 \| |

B: at baseline; 3M: after 3 months of F-U; NA: non-applicable

Group 2:

|  | **P1**  **B vs at 3M** | **P2**  **B vs at 3M** | **P3**  **B vs at 3M** | **P4**  **B vs at 3M** | **P5**  **B vs at 3M** |
| --- | --- | --- | --- | --- | --- |
| **Pre vs post**   - **Fasting** - **30’** - **60’** - **120’** | \| 174,3 vs 184,5 \| \| --- \| \| 183,9 vs 153,0 \| \| 179,1 vs 150,8 \| \| 159,3 vs 154,3 \| | \| 225,8 vs 177,7 \| \| --- \| \| 212,1 vs 151,4 \| \| 204,8 vs 163,0 \| \| 189,1 vs 163,6 \| | \| 162,4 vs 183,9 \| \| --- \| \| 157,5 vs 220,1 \| \| 169,1 vs 190,2 \| \| 159,1 vs 176,9 \| | \| 201,3 vs NA \| \| --- \| \| 190,4 vs NA \| \| 168,0 vs NA \| \| 158,1 vs NA \| | \| 153, 2 vs 150,4 \| \| --- \| \| 164,4 vs 182,2 \| \| NA vs 163,0 \| \| NA vs 147,9 \| |

B: at baseline; 3M: after 3 months of F-U; NA: non-applicable

Group 3:

|  | **P1**  **B vs at 3M** | **P2**  **B vs at 3M** | **P3**  **B vs at 3M** |
| --- | --- | --- | --- |
| **Pre vs post**   - **Fasting** - **30’** - **60’** - **120’** | \| 207,4 vs NA \| \| --- \| \| 150,2 vs NA \| \| 145,1 vs NA \| \| 152,6 vs NA \| | \| NA vs 230,8 \| \| --- \| \| NA vs 226,5 \| \| NA vs 214,4 \| \| NA vs 216,6 \| | \| NA vs 187,2 \| \| --- \| \| NA vs 215,9 \| \| NA vs 207,4 \| \| NA vs 187,0 \| |

B: at baseline; 3M: after 3 months of F-U; NA: non-applicable

Group 4:

|  | **P1**  **B vs at 3M** | **P2**  **B vs at 3M** | **P3**  **B vs at 3M** | **P4**  **B vs at 3M** |
| --- | --- | --- | --- | --- |
| **Pre vs post**   - **Fasting** - **30’** - **60’** - **120’** | \| NA vs 169,7 \| \| --- \| \| NA vs 138,2 \| \| NA vs 135,1 \| \| NA vs 134,6 \| | \| 227,3 vs 167,7 \| \| --- \| \| 222,3 vs 171,1 \| \| 214,4 vs 172,7 \| \| 224,6 vs 186,7 \| | \| 217,5 vs 166,6 \| \| --- \| \| 202,9 vs 150,8 \| \| 215,4 vs 151,5 \| \| 214,4 vs 154,1 \| | \| 178,0 vs NA \| \| --- \| \| 183,0 vs NA \| \| 196,5 vs NA \| \| 241,5 vs NA \| |

B: at baseline; 3M: after 3 months of F-U; NA: non-applicable

Table 4S: Xylose evaluation.

Group 1:

|  | **P1**  **B vs at 3M** | **P2**  **B vs at 3M** | **P3**  **B vs at 3M** | **P4**  **B vs at 3M** | **P5**  **B vs at 3M** | **P6**  **B vs at 3M** | **P7**  **B vs at 3M** |
| --- | --- | --- | --- | --- | --- | --- | --- |
| **Pre vs post**   - **Fasting** - **30’** - **60’** - **120’** | \| NA vs NA \| \| --- \| \| 0,0 vs NA \| \| 46,0 vs NA \| \| 24,8 vs NA \| | \| NA vs NA \| \| --- \| \| NA vs NA \| \| 10,6 vs NA \| \| 46,0 vs NA \| | \| 0,0 vs NA \| \| --- \| \| 74,3 vs NA \| \| 81,4 vs NA \| \| 17,7 vs NA \| | \| NA vs 0,0 \| \| --- \| \| NA vs 10,6 \| \| NA vs 0,0 \| \| NA vs 0,0 \| | \| NA vs 122,1 \| \| --- \| \| NA vs NA \| \| NA vs NA \| \| NA vs 0 \| | \| 0 vs 74,3 \| \| --- \| \| 24,8 vs 53,1 \| \| NA vs NA \| \| NA vs 81,4 \| | \| 38,9 vs NA \| \| --- \| \| 0,0 vs NA \| \| 31,8 vs NA \| \| NA vs NA \| |

B: at baseline; 3M: after 3 months of F-U; NA: non-applicable

Group 2:

|  | **P1**  **B vs at 3M** | **P2**  **B vs at 3M** | **P3**  **B vs at 3M** | **P4**  **B vs at 3M** | **P5**  **B vs at 3M** |
| --- | --- | --- | --- | --- | --- |
| **Pre vs post**   - **Fasting** - **30’** - **60’** - **120’** | \| NA vs NA \| \| --- \| \| NA vs NA \| \| 24,8 vs NA \| \| 102,6 vs NA \| | \| 0,0 vs NA \| \| --- \| \| 74,3 vs 17,7 \| \| 67,2 vs 24,8 \| \| 53,1 vs 38,9 \| | \| NA vs NA \| \| --- \| \| NA vs NA \| \| NA vs NA \| \| NA vs NA \| | \| 30,1 vs NA \| \| --- \| \| 0,0 vs NA \| \| 5,3 vs NA \| \| NA vs NA \| | \| NA vs 31,8 \| \| --- \| \| 17,7 vs 0 \| \| NA vs 31,8 \| \| NA vs NA \| |

B: at baseline; 3M: after 3 months of F-U; NA: non-applicable

Group 3:

|  | **P1**  **B vs at 3M** | **P2**  **B vs at 3M** | **P3**  **B vs at 3M** |
| --- | --- | --- | --- |
| **Pre vs post**   - **Fasting** - **30’** - **60’** - **120’** | \| NA vs NA \| \| --- \| \| NA vs NA \| \| NA vs NA \| \| NA vs NA \| | \| NA vs 5,3 \| \| --- \| \| NA vs 44,2 \| \| NA vs 0 \| \| NA vs 30,1 \| | \| NA vs 0 \| \| --- \| \| NA vs 19,5 \| \| NA vs 0 \| \| NA vs 8,8 \| |

B: at baseline; 3M: after 3 months of F-U; NA: non-applicable

Group 4:

|  | **P1**  **B vs at 3M** | **P2**  **B vs at 3M** | **P3**  **B vs at 3M** | **P4**  **B vs at 3M** |
| --- | --- | --- | --- | --- |
| **Pre vs post**   - **Fasting** - **30’** - **60’** - **120’** | \| NA vs NA \| \| --- \| \| NA vs NA \| \| NA vs NA \| \| NA vs NA \| | \| 138,0 vs 159,2 \| \| --- \| \| 81,4 vs 145,1 \| \| 123,8 vs 95,5 \| \| 145,1 vs 102,6 \| | \| 10,6 vs 17,7 \| \| --- \| \| NA vs 102,6 \| \| NA vs 95,5 \| \| NA vs 88,4 \| \|  \| \|  \| | \| NA vs NA \| \| --- \| \| 12,4 vs NA \| \| NA vs NA \| \| NA vs NA \| |

B: at baseline; 3M: after 3 months of F-U; NA: non-applicable

Table 5S: FGF-19 valuation.

Group 1:

|  | **P1**  **B vs at 3M** | **P2**  **B vs at 3M** | **P3**  **B vs at 3M** | **P4**  **B vs at 3M** | **P5**  **B vs at 3M** | **P6**  **B vs at 3M** | **P7**  **B vs at 3M** |
| --- | --- | --- | --- | --- | --- | --- | --- |
| **Pre vs post**   - **Fasting** - **30’** - **60’** - **120’** | \| NA vs NA \| \| --- \| \| NA vs NA \| \| NA vs NA \| \| NA vs NA \| | \| NA vs NA \| \| --- \| \| NA vs NA \| \| NA vs NA \| \| NA vs NA \| | \| NA vs NA \| \| --- \| \| NA vs NA \| \| NA vs NA \| \| NA vs NA \| | \| 5,37 vs NA \| \| --- \| \| 1,95 vs NA \| \| 0,65 vs NA \| \| NA vs NA \| | \| NA vs NA \| \| --- \| \| NA vs NA \| \| NA vs NA \| \| NA vs NA \| | \| NA vs NA \| \| --- \| \| NA vs NA \| \| NA vs NA \| \| NA vs NA \| | \| NA vs NA \| \| --- \| \| NA vs NA \| \| NA vs NA \| \| NA vs NA \| |

B: at baseline; 3M: after 3 months of F-U; NA: non-applicable

Group 2:

|  | **P1**  **B vs at 3M** | **P2**  **B vs at 3M** | **P3**  **B vs at 3M** | **P4**  **B vs at 3M** | **P5**  **B vs at 3M** |
| --- | --- | --- | --- | --- | --- |
| **Pre vs post**   - **Fasting** - **30’** - **60’** - **120’** | \| 0,48 vs NA \| \| --- \| \| 2,39 vs NA \| \| 2,97 vs NA \| \| 1,42 vs NA \| \|  \| | \| NA vs NA \| \| --- \| \| NA vs NA \| \| NA vs NA \| \| NA vs NA \| | \| NA vs NA \| \| --- \| \| NA vs NA \| \| NA vs NA \| \| NA vs NA \| \|  \| | \| 5,37 vs NA \| \| --- \| \| 1,95 vs NA \| \| 0,65 vs NA \| \| NA vs NA \| | \| NA vs NA \| \| --- \| \| NA vs NA \| \| NA vs NA \| \| NA vs NA \| |

B: at baseline; 3M: after 3 months of F-U; NA: non-applicable

Group 3:

|  | **P1**  **B vs at 3M** | **P2**  **B vs at 3M** | **P3**  **B vs at 3M** |
| --- | --- | --- | --- |
| **Pre vs post**   - **Fasting** - **30’** - **60’** - **120’** | \| NA vs NA \| \| --- \| \| NA vs NA \| \| NA vs NA \| \| NA vs NA \| | \| NA vs NA \| \| --- \| \| NA vs 1,84 \| \| NA vs 0,65 \| \| NA vs 1,31 \| | \| NA vs NA \| \| --- \| \| NA vs 1,31 \| \| NA vs 0,74 \| \| NA vs NA \| |

B: at baseline; 3M: after 3 months of F-U; NA: non-applicable

Group 4:

|  | **P1**  **B vs at 3M** | **P2**  **B vs at 3M** | **P3**  **B vs at 3M** | **P4**  **B vs at 3M** |
| --- | --- | --- | --- | --- |
| **Pre vs post**   - **Fasting** - **30’** - **60’** - **120’** | \| NA vs 1,84 \| \| --- \| \| NA vs 1,52 \| \| NA vs 1,02 \| \| NA vs 0,74 \| | \| NA vs NA \| \| --- \| \| NA vs NA \| \| NA vs NA \| \| NA vs NA \| | \| NA vs NA \| \| --- \| \| NA vs NA \| \| NA vs NA \| \| NA vs NA \| \|  \| \|  \| | \| NA vs NA \| \| --- \| \| NA vs NA \| \| NA vs NA \| \| NA vs NA \| |

B: at baseline; 3M: after 3 months of F-U; NA: non-applicable

Table 6S: FGF-21 evaluation. Group 1:

|  | **P1**  **B vs at 3M** | **P2**  **B vs at 3M** | **P3**  **B vs at 3M** | **P4**  **B vs at 3M** | **P5**  **B vs at 3M** | **P6**  **B vs at 3M** | **P7**  **B vs at 3M** |
| --- | --- | --- | --- | --- | --- | --- | --- |
| **Pre vs post**   - **Fasting** - **30’** - **60’** - **120’** | \| NA vs NA \| \| --- \| \| NA vs NA \| \| NA vs NA \| \| NA vs NA \| | \| NA vs NA \| \| --- \| \| NA vs NA \| \| 5,5 vs NA \| \| NA vs NA \| | \| NA vs NA \| \| --- \| \| NA vs NA \| \| NA vs NA \| \| NA vs NA \| | \| NA vs NA \| \| --- \| \| NA vs NA \| \| NA vs NA \| \| NA vs NA \| | \| NA vs NA \| \| --- \| \| NA vs NA \| \| NA vs NA \| \| NA vs NA \| | \| NA vs NA \| \| --- \| \| NA vs NA \| \| NA vs NA \| \| NA vs NA \| | \| NA vs NA \| \| --- \| \| NA vs NA \| \| NA vs NA \| \| NA vs NA \| |

B: at baseline; 3M: after 3 months of F-U; NA: non-applicable

Group 2:

|  | **P1**  **B vs at 3M** | **P2**  **B vs at 3M** | **P3**  **B vs at 3M** | **P4**  **B vs at 3M** | **P5**  **B vs at 3M** |
| --- | --- | --- | --- | --- | --- |
| **Pre vs post**   - **Fasting** - **30’** - **60’** - **120’** | \| NA vs 10,04 \| \| --- \| \| NA vs 12,56 \| \| NA vs 7,11 \| \| NA vs NA \| \|  \| \|  \| | \| NA vs 2,24 \| \| --- \| \| NA vs NA \| \| NA vs 7,99 \| \| NA vs 65,59 \| \|  \| \|  \| \|  \| | \| NA vs 19,79 \| \| --- \| \| 0,68 vs NA \| \| 0,44 vs 7,11 \| \| NA vs NA \| \|  \| \|  \| | \| NA vs NA \| \| --- \| \| NA vs NA \| \| NA vs NA \| \| NA vs NA \| | \| NA vs NA \| \| --- \| \| NA vs NA \| \| NA vs NA \| \| NA vs NA \| \|  \| |

B: at baseline; 3M: after 3 months of F-U; NA: non-applicable

Group 3:

|  | **P1**  **B vs at 3M** | **P2**  **B vs at 3M** | **P3**  **B vs at 3M** |
| --- | --- | --- | --- |
| **Pre vs post**   - **Fasting** - **30’** - **60’** - **120’** | \| NA vs NA \| \| --- \| \| NA vs NA \| \| NA vs NA \| \| NA vs NA \| | \| NA vs NA \| \| --- \| \| NA vs NA \| \| NA vs NA \| \| NA vs NA \| | \| NA vs NA \| \| --- \| \| NA vs NA \| \| NA vs NA \| \| NA vs NA \| |

B: at baseline; 3M: after 3 months of F-U; NA: non-applicable

Group 4:

|  | **P1**  **B vs at 3M** | **P2**  **B vs at 3M** | **P3**  **B vs at 3M** | **P4**  **B vs at 3M** |
| --- | --- | --- | --- | --- |
| **Pre vs post**   - **Fasting** - **30’** - **60’** - **120’** | \| NA vs NA \| \| --- \| \| NA vs NA \| \| NA vs NA \| \| NA vs NA \| | \| NA vs NA \| \| --- \| \| NA vs NA \| \| NA vs NA \| \| NA vs NA \| | \| NA vs NA \| \| --- \| \| NA vs NA \| \| NA vs NA \| \| NA vs NA \| \|  \| \|  \| | \| NA vs NA \| \| --- \| \| NA vs NA \| \| NA vs NA \| \| NA vs NA \| |

B: at baseline; 3M: after 3 months of F-U; NA: non-applicable

Table 7S: GLP-1 Evaluation.

Group 1:

|  | **P1**  **B vs at 3M** | **P2**  **B vs at 3M** | **P3**  **B vs at 3M** | **P4**  **B vs at 3M** | **P5**  **B vs at 3M** | **P6**  **B vs at 3M** | **P7**  **B vs at 3M** |
| --- | --- | --- | --- | --- | --- | --- | --- |
| **Pre vs post**   - **Fasting** - **30’** - **60’** - **120’** | \| NA vs NA \| \| --- \| \| NA vs NA \| \| NA vs NA \| \| NA vs NA \| | \| NA vs NA \| \| --- \| \| NA vs NA \| \| NA vs NA \| \| NA vs NA \| | \| NA vs NA \| \| --- \| \| NA vs NA \| \| NA vs NA \| \| NA vs NA \| | \| NA vs NA \| \| --- \| \| NA vs NA \| \| NA vs NA \| \| NA vs NA \| | \| NA vs NA \| \| --- \| \| NA vs NA \| \| NA vs NA \| \| NA vs NA \| | \| NA vs NA \| \| --- \| \| NA vs NA \| \| NA vs NA \| \| NA vs NA \| | \| NA vs NA \| \| --- \| \| NA vs NA \| \| NA vs NA \| \| NA vs NA \| |

B: at baseline; 3M: after 3 months of F-U; NA: non-applicable

Group 2:

|  | **P1**  **B vs at 3M** | **P2**  **B vs at 3M** | **P3**  **B vs at 3M** | **P4**  **B vs at 3M** | **P5**  **B vs at 3M** |
| --- | --- | --- | --- | --- | --- |
| **Pre vs post**   - **Fasting** - **30’** - **60’** - **120’** | \| NA vs NA \| \| --- \| \| NA vs NA \| \| NA vs NA \| \| NA vs NA \| | \| NA vs NA \| \| --- \| \| NA vs NA \| \| NA vs NA \| \| NA vs NA \| | \| NA vs NA \| \| --- \| \| NA vs NA \| \| NA vs NA \| \| NA vs NA \| | \| NA vs NA \| \| --- \| \| NA vs NA \| \| NA vs NA \| \| NA vs NA \| | \| NA vs NA \| \| --- \| \| NA vs NA \| \| NA vs NA \| \| NA vs NA \| |

B: at baseline; 3M: after 3 months of F-U; NA: non-applicable

Group 3:

|  | **P1**  **B vs at 3M** | **P2**  **B vs at 3M** | **P3**  **B vs at 3M** |
| --- | --- | --- | --- |
| **Pre vs post**   - **Fasting** - **30’** - **60’** - **120’** | \| NA vs NA \| \| --- \| \| NA vs NA \| \| NA vs NA \| \| NA vs NA \| | \| NA vs NA \| \| --- \| \| NA vs NA \| \| NA vs NA \| \| NA vs NA \| | \| NA vs NA \| \| --- \| \| NA vs NA \| \| NA vs NA \| \| NA vs NA \| |

B: at baseline; 3M: after 3 months of F-U; NA: non-applicable

Group 4:

|  | **P1**  **B vs at 3M** | **P2**  **B vs at 3M** | **P3**  **B vs at 3M** | **P4**  **B vs at 3M** |
| --- | --- | --- | --- | --- |
| **Pre vs post**   - **Fasting** - **30’** - **60’** - **120’** | \| NA vs NA \| \| --- \| \| NA vs NA \| \| NA vs NA \| \| NA vs NA \| | \| NA vs NA \| \| --- \| \| NA vs NA \| \| NA vs NA \| \| NA vs NA \| | \| NA vs NA \| \| --- \| \| NA vs NA \| \| NA vs NA \| \| NA vs NA \| \|  \| \|  \| | \| NA vs NA \| \| --- \| \| NA vs NA \| \| NA vs NA \| \| NA vs NA \| |

B: at baseline; 3M: after 3 months of F-U; NA: non-applicable
